# Polyoxometalates: metallodrug agents for combating amyloid aggregation

**DOI:** 10.1093/nsr/nwae226

**Published:** 2024-06-28

**Authors:** Mengmeng Ma, Zhenqi Liu, Huisi Zhao, Haochen Zhang, Jinsong Ren, Xiaogang Qu

**Affiliations:** Laboratory of Chemical Biology and State Key Laboratory of Rare Earth Resource Utilization, Changchun Institute of Applied Chemistry, Chinese Academy of Sciences, Changchun 130022, China; School of Applied Chemistry and Engineering, University of Science and Technology of China, Hefei 230026, China; Laboratory of Chemical Biology and State Key Laboratory of Rare Earth Resource Utilization, Changchun Institute of Applied Chemistry, Chinese Academy of Sciences, Changchun 130022, China; School of Applied Chemistry and Engineering, University of Science and Technology of China, Hefei 230026, China; Laboratory of Chemical Biology and State Key Laboratory of Rare Earth Resource Utilization, Changchun Institute of Applied Chemistry, Chinese Academy of Sciences, Changchun 130022, China; School of Applied Chemistry and Engineering, University of Science and Technology of China, Hefei 230026, China; Laboratory of Chemical Biology and State Key Laboratory of Rare Earth Resource Utilization, Changchun Institute of Applied Chemistry, Chinese Academy of Sciences, Changchun 130022, China; School of Applied Chemistry and Engineering, University of Science and Technology of China, Hefei 230026, China; Laboratory of Chemical Biology and State Key Laboratory of Rare Earth Resource Utilization, Changchun Institute of Applied Chemistry, Chinese Academy of Sciences, Changchun 130022, China; School of Applied Chemistry and Engineering, University of Science and Technology of China, Hefei 230026, China; Laboratory of Chemical Biology and State Key Laboratory of Rare Earth Resource Utilization, Changchun Institute of Applied Chemistry, Chinese Academy of Sciences, Changchun 130022, China; School of Applied Chemistry and Engineering, University of Science and Technology of China, Hefei 230026, China

**Keywords:** amyloid inhibition, metallodrug, polyoxometalate, amyloid-β, Alzheimer's disease

## Abstract

Alzheimer's disease (AD) is a devastating neurodegenerative disease that affects ∼50 million people globally. The accumulation of amyloid-β (Aβ) plaques, a predominant pathological feature of AD, plays a crucial role in AD pathogenesis. In this respect, Aβ has been regarded as a highly promising therapeutic target for AD treatment. Polyoxometalates (POMs) are a novel class of metallodrugs being developed as modulators of Aβ aggregation, owing to their negative charge, polarity, and three-dimensional structure. Unlike traditional discrete inorganic complexes, POMs contain tens to hundreds of metal atoms, showcasing remarkable tunability and diversity in nuclearities, sizes, and shapes. The easily adjustable and structurally variable nature of POMs allows for their favorable interactions with Aβ. This mini-review presents a balanced overview of recent progress in using POMs to mitigate amyloidosis. Clear correlations between anti-amyloid activities and structural features of POMs are also elaborated in detail. Finally, we discuss the current challenges and future prospects of POMs in combating AD.

## Teaser

This review provides new insights into design and screening of polyoxometalates (POMs) to inhibit amyloidosis, elaborate the correlations between anti-amyloid activities and structural features of POMs, and further highlights challenges and future prospects of POMs in combating Alzheimer's disease.

## INTRODUCTION

Alzheimer's disease (AD), a neurodegenerative disorder associated with aging, affects ∼50 million people and begets an ongoing socio-economic burden globally [1]. The dominating pathological hallmarks of AD are extracellular amyloid-β (Aβ) plaques and intracellular neurofibrillary tangles [2,3]. Although the detailed etiology of AD remains unclear, the mainstream belief now is that the widespread accumulation of Aβ peptide in the brain is an initial and pivotal event in the progression of AD, further eliciting a series of detrimental cascades, including tau pathology, chronic inflammation, and cognitive malfunction [4–7]. Recently, three anti-amyloid monoclonal antibodies, aducanumab, lecanemab, and donanemab, have been approved for AD therapy by the U.S. Food and Drug Administration (FDA). These antibodies can clear Aβ plaques and postpone cognitive decline in early-stage AD patients, validating the therapeutic strategy of targeting Aβ [8]. Therefore, modulation of Aβ aggregation is perceived as a promising strategy in the fight against AD.

**Figure 1.**
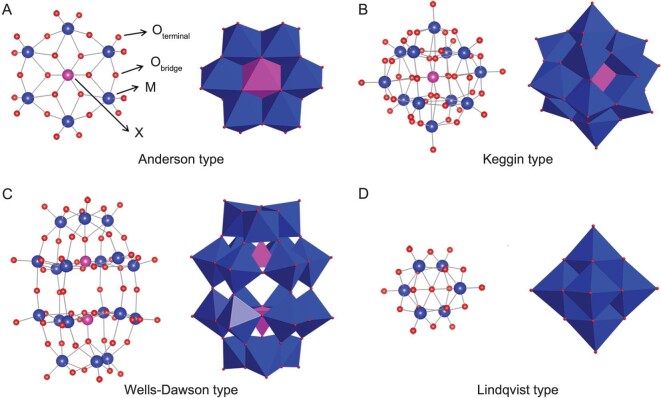
Ball-and-stick and polyhedral representation of four most common types of POMs. (A) Anderson structure [XM_6_O_24_]^n−^, (B) Keggin structure [XM_12_O_40_]^n−^, (C) Wells-Dawson structure [X_2_M_18_O_62_]^n−^, (D) Lindqvist structure [M_6_O_19_]^n−^. M: addenda metal atoms; X: heteroatoms; O_terminal_: terminal oxygen atoms; O_bridge_: bridge oxygen atoms.

**Figure 2.**
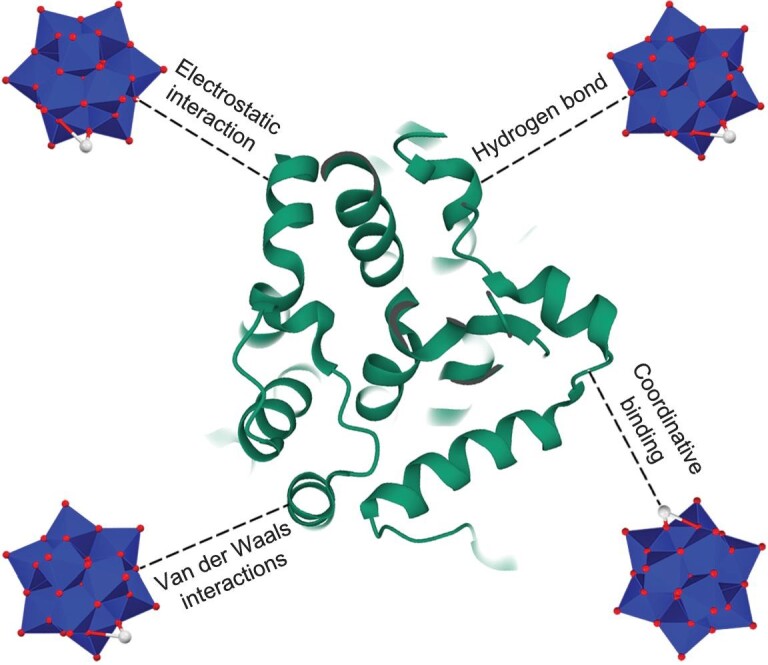
Overview of the common POM-protein interactions, including electrostatic interaction between POMs and proteins containing positively charged functional groups, hydrogen bonding between POMs and proteins containing hydroxyl functional groups, van der Waals interaction between POMs and proteins containing methyl functional groups, and coordinative binding between substituted metal ions in POMs and oxygen or nitrogen atoms in proteins.

Numerous metal compounds have been developed as metallodrug agents targeting various diseases, including diabetes, cardiovascular diseases, and cancer [9–12]. Lately, metallodrugs have gained considerable interest as potential modulators of Aβ aggregation, due to their remarkable physicochemical properties such as diverse coordination geometries and multiple metal center oxidation states [13–20]. These metallodrugs are capable of disrupting Aβ aggregation and alleviating Aβ-related neurotoxicity through both direct and indirect interactions with Aβ, including electrostatic attraction, coordination, π–π stacking, oxidation, and hydrolysis. Polyoxometalates (POMs), a burgeoning class of metallodrugs, are often described as discrete clusters of early transition metal oxides, with the central metal ions typically in their highest oxidation state [21–24]. POMs can be divided into two primary groups: iso-POMs [M*_x_*O*_y_*]*^n^*^−^ and hetero-POMs [X*_x_*M*_y_*O*_z_*]*^n^*^−^ (where M = V, Mo, W, Nb, Ta; X = B, As, Si, P, Ge, etc.) [25,26]. Both iso-POMs and hetero-POMs generally consist of octahedral [MO_6_] synthons interconnected by bridging oxo atoms. While numerous POM structures have been identified to date, most studied POMs fall into one of the four prevalent POM archetypes (Anderson-, Keggin-, Wells-Dawson-, and Lindqvist-type structures) [25,27], as depicted in Fig. 1. POMs hold significant advantages over traditional discrete inorganic clusters, as they can be easily derived from cost-effective inorganic salts and provide a broad spectrum of structures with diverse elemental compositions, geometries, and solution stability [28–33]. Crucially, their associated chemical properties, such as redox potential, acidity, polarity, and surface charge distribution, can be precisely tailored to specific needs by adjusting factors like composition, size, shape, and counterion [34,35]. These outstanding features gift POMs with multifarious bioactivities in the fight against cancer, viruses, diabetes, and bacteria [27,31,33,36–39]. The pharmacological and biological attributes of POMs are largely ascribed to their interactions with proteins [33,40,41]. Exhilaratingly, several studies have examined the interactions between POMs and proteins [41–43]. As revealed by various techniques such as X-ray crystallography, computational modeling, and isothermal titration calorimetry, POMs typically form multiple types of interactions with proteins, including electrostatic, hydrogen bonding, van der Waals, and coordination interactions (Fig. 2) [41–43]. Electrostatic attractions are the dominant forces in POM-protein interactions, due to the polyanionic and oxygen-rich nature of POMs. Specifically, POMs tend to preferentially interact with protein regions containing positively charged functional groups. Alongside electrostatic interactions, noncovalent binding of POMs with proteins is predominantly governed by hydrogen bonds. Instead of outlining the progress on specific protein interactions with POMs, Parac-Vogt *et al.* recently overviewed the general principle on POM-protein interactions, guiding the field development [43]. Such direct interactions of POMs with proteins enlightened us to explore their effects on Aβ folding and conformation. Our first preliminary works focused on the modulation effect of POMs toward Aβ aggregation [44]. Unsurprisingly, the screening results indicated that these typical POM compounds, including Wells-Dawson and Keggin structures, bound strongly to Aβ and hindered Aβ aggregation [44]. The nascent POMs-based strategy for modulating Aβ aggregation may shed light toward the design and screening of cost-effective metallodrugs to treat devastating AD.

**Figure 3.**
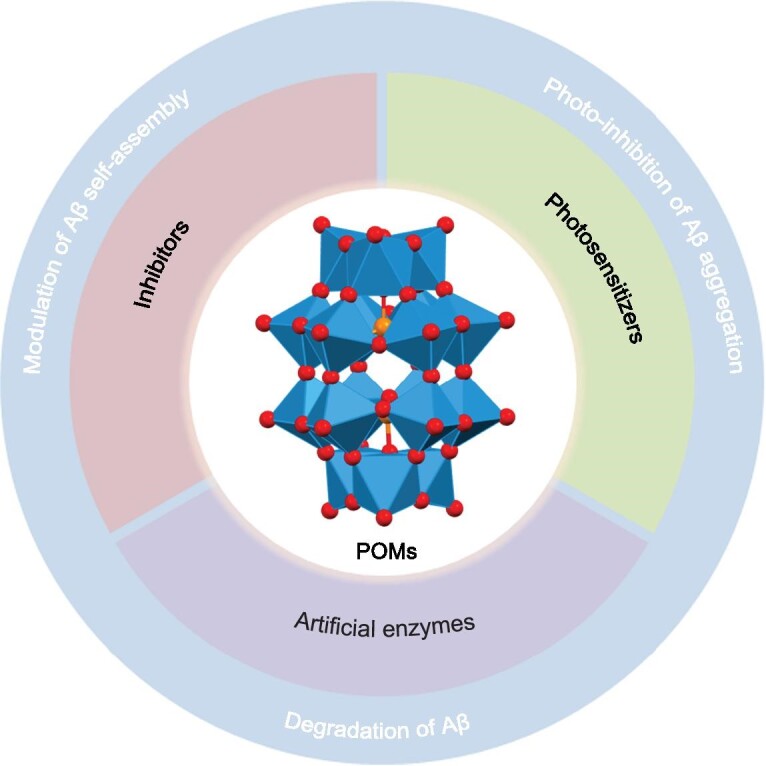
Schematic representation of anti-Aβ activity of POMs.

In this review, we delineate and classify the advances of POMs based on their means of intervening with the aggregation of Aβ and site-directed modification of Aβ (Fig. 3). The discussion mainly focuses on their design, working mechanisms toward the aggregation of Aβ, and corresponding applications in AD treatment. At the end, the scientific opportunities and challenges for future advancement in the field are also discussed.

## POMS AS INHIBITORS OF Aβ AGGREGATION

### Modulate A**β** aggregation through non-covalent interactions

#### Anti-amyloid activity of POMs

**Figure 4.**
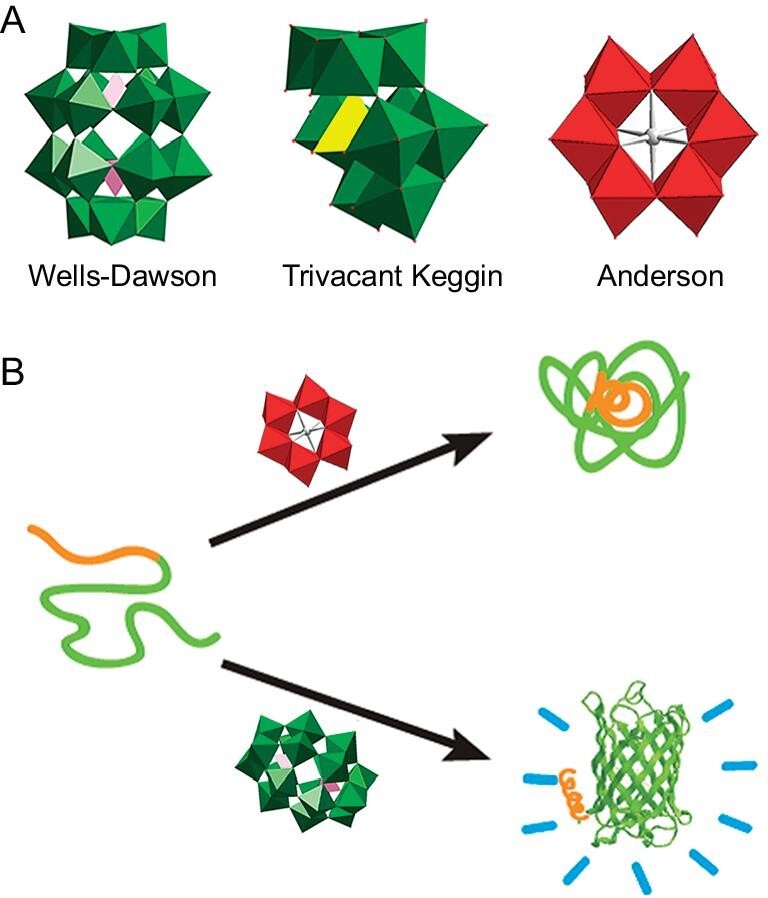
(A) Representation structures of typical POMs. (B) Different types of POMs as amyloid aggregation inhibitors (unlike Anderson POMs (red), the Wells-Dawson POMs (green) could inhibit Aβ aggregation). Adapted with permission from ref. [44], Wiley.

With the in-depth understanding of POM-protein interactions, our group demonstrated that POMs could efficiently block Aβ_40_ conformational changes and redirect Aβ_40_ aggregation into off-pathway, unstructured aggregates (Fig. 4) [44]. The inhibition activity and selectivity of POMs resulted from size-specific electrostatic interactions between POMs and the cationic cluster His13-Lys16 of Aβ, which primarily depends on the size and charge of POMs. The largest Wells-Dawson POMs K_8_[P_2_CoW_17_O_61_] were most efficient in inhibition of Aβ aggregation (half maximal inhibitory concentration (IC_50_) = 16.68 μM). Keggin POMs K_8_[b-SiW_11_O_39_] exhibited a moderate inhibitory effect (IC_50_ = 39.02 μM). Anderson POMs Na_5_[IMo_6_O_24_] were inactive. Digestion assays with trypsin and competition experiments with 4,4′-bis(1-anilinonaphthalene 8-sulfonate) revealed that polyanionic POMs bound specifically to the positively charged motif of Aβ (His13-Lys16). This work established the firm foundation for POMs as a new class of Aβ inhibitors. Zhou *et al.* also reported that the wheel-shaped structure POM (P_5_W_30_) could interact with Aβ peptides and suppress their fibrillization [45]. In addition, Liu's group found that the spherical shape of POMs exhibited strong inhibitory effects against Aβ aggregation [46]. Intriguingly, they also prevented Cu^2+^- or Zn^2+^-induced Aβ aggregation and blocked Aβ-mediated neurotoxicity.

**Figure 5.**
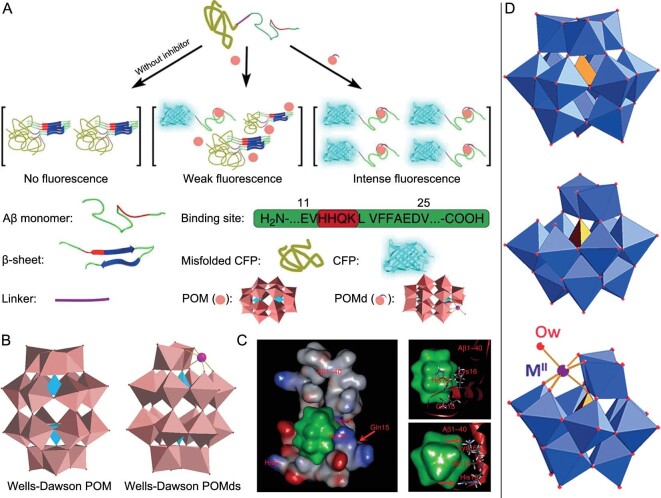
(A) Schematic illustration of the CFP-fusion expression system for screening Aβ inhibitors. (B) Structural representation of Wells-Dawson POM, and Wells-Dawson POMds containing histidine–chelating transition-metal ions. (C) The binding mode of POM to Aβ obtained from molecular dynamic simulations. (D) Structural representation of K_4_[α-SiW_12_O_40_], K_8_[α-SiW_11_O_39_], and [α-SiW_11_O_39_M^II^(OH_2_)]_6_ (Ow = water molecule, M^II^ = d-metal dication). Adapted with permission from ref. [47], Springer Nature Ltd; ref. [48], Royal Society of Chemistry.

Previous studies have shown that the distinctive interaction between POMs and Aβ largely relies on electrostatic attractions. Although the POMs-Aβ electrostatic interactions are robust, the binding affinity of POMs to Aβ could still be improved. Notably, this cationic His13-Lys16 (HHQK) cluster in Aβ comprises two adjacent histidine amino acids, which are able to chelate transition metal ions. Consequently, our group constructed a variety of transition-metal (Mn, Cu, Fe, Co, Ni)-substituted Wells-Dawson POMs (POMds) [47]. Engineering of a high-throughput screening approach based on the cyan fluorescent protein (CFP)-fusion expression system, we demonstrated that POMds exhibited stronger Aβ binding affinity and superior Aβ inhibitory effect compared with the parent Wells-Dawson POMs (Fig. 5A and B). This was ascribed to the robust coordinating interactions between POMds and His13/His14 (Fig. 5C). In addition, POMds could reduce Aβ-heme peroxidase-like activity. More intriguingly, increased accumulation of POMds in the brain was observed, peaking at 10 minutes after injection, which suggested their potential ability to pass through the blood-brain barrier (BBB). Moreover, the level of POMds in the brain began to decrease after 10 minutes post-injection and returned to initial levels after 48 hours under the experimental conditions. These results indicated that POMds could be cleared from the brain over time, avoiding long-term toxicity. Although POMds could cross the BBB, just a small fraction of the injected dose actually reached brain tissues due to their suboptimal BBB permeability and selectivity. Furthermore, Hureau's group found that the lacunary POMs with Keggin structure removed Cu^2+^ ions bound to Aβ, stopped Aβ-Cu^2+^ complex-induced reactive oxygen species (ROS) production, and modified Aβ aggregation (Fig. 5D) [48].

#### Anti-amyloid activity of POMs-organic hybrids

**Figure 6.**
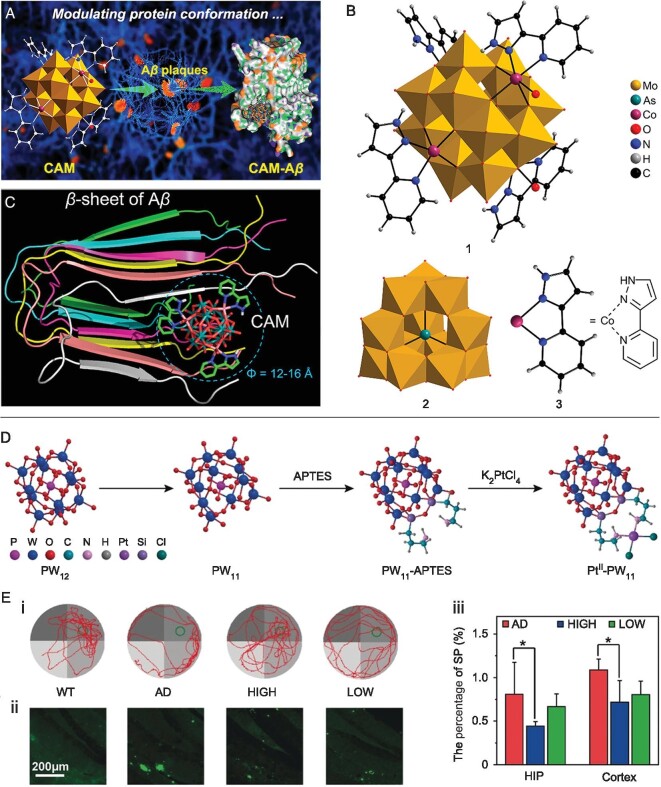
(A) Schematic illustration of CAM used for the modulation of Aβ aggregation. (B) Structural representation of CAM (1), ε-Keggin (HAsMo_12_O_40_)^8−^ unit (2), ε-Keggin (HAsMo_12_O_40_)^8−^ unit, and CoL^2+^ coordination unit (3). (C) The space-filling mode of CAM-Aβ interactions. (D) The synthetic route of Pt-substituted POM (Pt^II^-PW_11_). (E) Pt-substituted POM rescues memory impairment in AD mouse models. (i) Typical swimming routes of mice in the probe trial. (ii) Immunofluorescence, and (iii) corresponding percentage of brain senile plaques among various groups. The terms ‘high’ and ‘low’ in the caption refer to AD mice treated with high dose (1.5 mg/mL) and low dose (1.0 mg/mL) Pt^II^-PW_11_, respectively. Adapted with permission from ref. [52], American Chemical Society; ref. [53], Wiley.

The POMs-Aβ interaction primarily relies on size-dependent electrostatic interactions. Therefore, pristine POMs still need improving their selectivity when binding to Aβ. The covalent/coordination modification of pristine POMs with bioactive ligands provides an opportunity to develop POMs-organic hybrids, which can increase selectivity and reduce toxicity [49,50]. Bioactive ligands refer to a kind of molecule that can specifically bind to a target biomolecule such as protein or receptor, and modulate its biological function or activity [51]. Currently, several bioactive ligands (e.g. small molecules, peptides, and antibodies) have been incorporated into POM hybrids to acheive safe and selective inhibition of Aβ aggregation. For example, Ma *et al.* employed an Aβ-targeting organometallic group CoL^2+^ [L = 2-(1Hpyrazol-3-yl)pyridine] for the hybridization with ε-Keggin (HAsMo_12_O_40_)^8−^ unit to develop a newly modified POM with excellent targeting ability toward Aβ (Fig. 6A and B) [52]. The organocobalt-substituted Keggin POM [CoL]_4_[H_2_O]_2_[HAsMo_12_O_40_] (abbreviated as CAM) selectively interacted with Aβ and disaggregated self-aggregated or metal-induced Aβ fibrils (Fig. 6C). This effect was attributed to the matching size of CAM and the cavity of Aβ aggregates, and the hydrogen-bonding interactions of CAM with amino residues in the cavity. As a result, CAM suppressed the production of ROS and decreased the synaptic toxicity of Aβ aggregates. Importantly, the well-designed CAM was lipophilic and penetrated the BBB. These results highlight the power of combining POMs with known Aβ-targeting ligands for more specific interactions.

Zhao *et al.* synthesized an organoplatinum-substituted Keggin POM for blocking Aβ aggregation (Fig. 6D) [53]. In comparison with the unmodified POM, the interaction of the Pt-substituted POM with Aβ was greatly improved and more specific, which was attributed to the synergistic effect of Pt^II^ ions with amino acid residues in Aβ. These multiple strong interactions endowed Pt-substituted POM with a remarkable inhibitory effect on Aβ fibrillation (IC_50_ = 0.62 μm). In cell-based experiments, the Pt-substituted POM significantly reduced Aβ aggregation-mediated neurotoxicity. Moreover, *in vivo* experiments verified the Pt-substituted POM decreased Aβ deposition and alleviated cognitive impairments in AD model mice without apparent systemic toxicity (Fig. 6E). These results emphasize the significance of structural modification of POMs to achieve enhanced and selective POMs-Aβ interactions.

**Figure 7.**
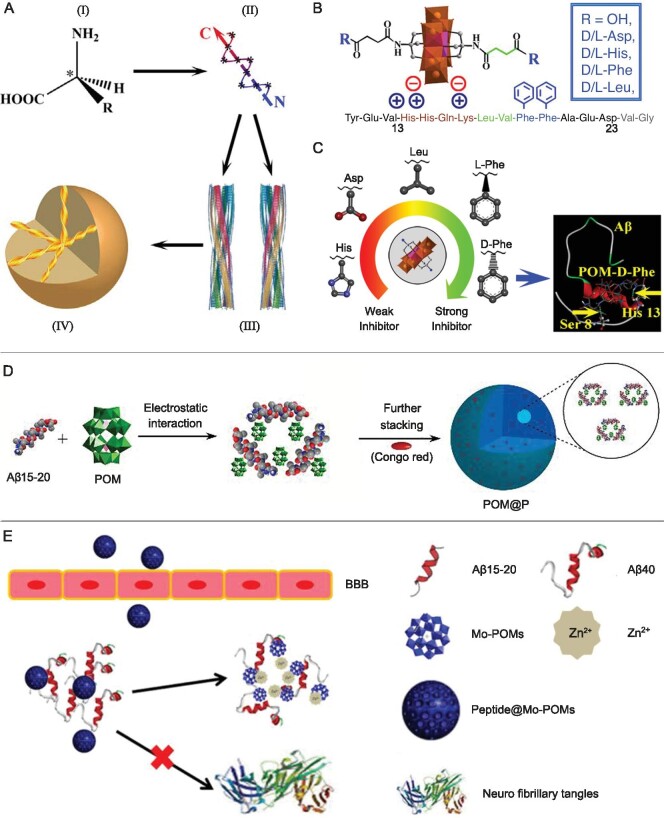
(A) Chirality at multiple size scales: (I) configurational chirality, (II) conformational chirality, (III) structural chirality, and (IV) object chirality. (B) Well-designed D-/L-amino acid-modified chiral POMs. (C) A sequence of D-/L-amino acid-modified chiral POMs as amyloid aggregation inhibitors (among them, D-Phe-modified chiral POMs exerted the most substantial suppression effect). And the energy-minimized average model of D-Phe-modified chiral POMs-Aβ interactions. (D) The synthetic route of POM@P hybrid nanocomposites. (E) The self-assembled Peptide@POMs could effectively cross the BBB and suppress Zn^2+^-mediated Aβ_40_ aggregation. Adapted with permission from ref. [54], American Chemical Society; ref. [60] and ref. [61], Wiley.

Chirality is the inherent characteristic of aggregated Aβ proteins, which can be classified into 4 levels (Fig. 7A) [54]. The first level is called ‘configurational chirality’, denoting the asymmetric arrangement of an atom with a set of ligands. The second level is known as ‘conformational chirality’, denoting the different helical conformation of Aβ. The third level is ‘structural chirality’, denoting the phase structure of Aβ fibrils. The fourth level is ‘object chirality’, which usually comes from the accumulation of helical single domains to form mesoscopic or macroscopic chiral objects. Many studies show that the chiral variations of amino acids are able to affect Aβ specificity and induce Aβ isomerization and epimerization, which are closely associated with AD dysfunction [55,56]. Therefore, the chirality strategy has garnered widespread attention in the design of amyloid inhibitors. Recently, our group prepared a range of chiral Anderson POMs modified with amino acids, including hydrophobic D-/L-Phe and Leu amino acids, negatively charged D-/L-Glu amino acids, and positively charged D-/L-His amino acids, for chirality-selected inhibition of Aβ fibrillation (Fig. 7B) [54]. According to fluorescence titration, isothermal titration calorimetry, circular dichroism, and ThT fluorescence assays, D-/L-Phe-modified chiral POMs exhibited higher binding affinity to Aβ and stronger inhibition effect against Aβ aggregation, compared to other D-/L-amino acid modified chiral POMs. Moreover, D-Phe-modified chiral POMs exerted a better suppression effect than the enantiomer L-Phe-modified chiral POMs because the Phe4, Ser8, His13, and Phe19 amino acid residues of Aβ preferred closing to D-Phe on the surface of POMs (Fig. 7C). More intriguingly, the chiral POMs crossed the BBB and prolonged the Caenorhabditis elegans CL2006 strain lifespan. Overall, these findings highlight the potential of enhanced POM-Aβ interactions through chiral ligand functionalization.

#### Anti-amyloid activity of POMs-based nanocomposites

POMs-Aβ interactions can also be tuned through supramolecular self-assembly to form POM-peptide hybrid nanocomposites. Many peptides/peptide mimetics possess strong anti-amyloid properties and are therefore an excellent option for hybridization with POMs to construct novel nanocomposites with improved biological activity [57–59]. Inspired by this phenomenon, Li *et al.* combined a Wells-Dawson POM K_8_[P_2_CoW_17_O_61_], with the well-known β-sheet breaker QKLVFF to self-assemble in nanospheres (abbreviated as POM@P) (Fig. 7D) [60]. The two-in-one POM@P nanocomposites exhibited both enhanced Aβ binding specificity and suppression of Aβ aggregation. Furthermore, by incorporating a probe molecule, Congo red, into the POM@P nanospheres, the nanocomposites could be utilized to detect the inhibitory effect of POM@P on Aβ aggregation in real time.

Liu *et al.* synthesized the Aβ-targeting peptide (Ac-QKLVFF-NH_2_)-modified Mo POM nanoparticles (denoted as Peptide@Mo-POMs) (Fig. 7E) [61]. After functionalization with Ac-QKLVFF-NH_2_, the Peptide@Mo-POMs could specifically target Aβ and dramatically enhance their ability to penetrate the BBB. *In vitro* experiments demonstrated that Peptide@Mo-POMs not only blocked Zn^2+^-mediated Aβ_40_ aggregation, but also decreased the Aβ_40_-induced neurotoxicity. Importantly, Peptide@Mo-POMs treatment improved the spatial cognitions of AD mice. Gao *et al.* also prepared a multifunctional AuNPs@POMD-pep [62], composed of Wells-Dawson POM (POMD), gold nanoparticles (AuNPs), and peptide (N-Ac-CLPFFD). The AuNPs@POMD-pep displayed synergistic effects on preventing Aβ aggregation and disassociating the formed Aβ fibrils. Moreover, through the use of AuNPs as BBB-transport vectors, the AuNPs@POMD-pep effectively crossed the BBB.

### Modulate A**β** aggregation through covalent modification

Most POMs-based inhibitors bind to Aβ through non-covalent interactions, which are susceptible to the surrounding environment and inherently weak. However, covalent interactions are not influenced by fluctuations in the surrounding environment and are chemically stable. It is well established that proteins undergo a variety of post-translational modifications (PTMs), such as covalent conjugations of glycans or phosphate groups to amino-acid side chains. Such covalent modifications can significantly change protein structures and activities. Aβ peptides have diverse PTMs that variously modulate both the aggregation states and bioactivities of Aβ [63]. Unfortunately, it is challenging to control PTM manually because of diverse subcellular localizations and intricate cellular signaling pathways. Thus, it is demanding to develop convenient and efficient chemical PTM agents.

**Figure 8.**
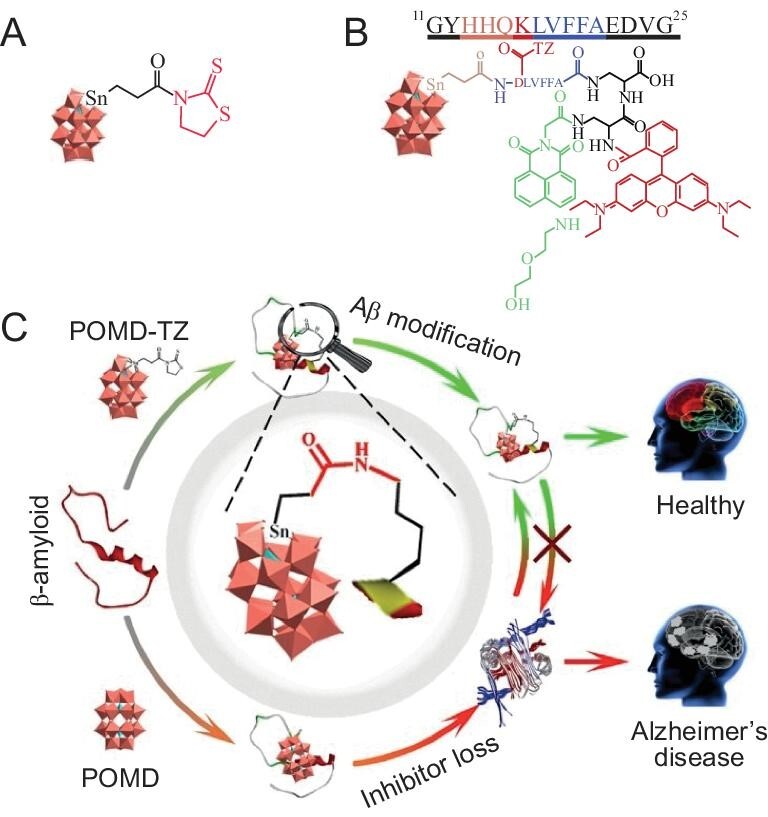
(A) Chemical structure of POMD-TZ. (B) Chemical structure of POMD-Tar-TZ-FRET. (C) Illustration of Aβ modification at the Lys16 site by POMD-TZ. Adapted with permission from ref. [64], Wiley.

For the first time, our group rationally designed a Wells-Dawson POMs-based PTM agent for site-directed chemical modification of Aβ (Fig. 8) [64]. Following the functionalization of POMs with thiazolidinethione (TZ), the resulting POMD-TZ served as a chemical PTM agent, covalently modifying Aβ at Lys16 residues (Fig. 8A). POMD-TZ exhibited a much stronger inhibitory effect on Aβ aggregation than the unmodified POMD. Moreover, *in vitro* biophysical and biochemical assays indicated that POMD-TZ site-specifically modified Aβ, modulated aggregation and mitigated Aβ aggregation-caused cytotoxicity. These results highlight the potential of covalent modification to construct new hybrid POMs with tailored bio-functionalities.

Next, we improved the therapeutic effect of chemical PTM agents in two ways: (i) to increase Aβ targeting ability, we added a TZ-modified aspartic acid (D) at the N-terminal of the Aβ targeting peptide LVFFA to synthesize POMD-Tar-TZ. (ii) To enable POM-TZ to differentiate between Aβ monomers and oligomers, we introduced a fluorescence resonance energy transfer (FRET) probe to produce POMD-Tar-TZ-FRET (Fig. 8B). The FRET between the naphthalimide-based fluorescent probe and rhodamine increased the sensitivity of Aβ oligomer detection. As a result, POMD-Tar-TZ-FRET not only covalently modified Aβ but also selectively detected and visualized Aβ oligomers.

## POMS AS PHOTOSENSITIZERS FOR PHOTO-INDUCED INHIBITION OF A**β** AGGREGATION

**Figure 9.**
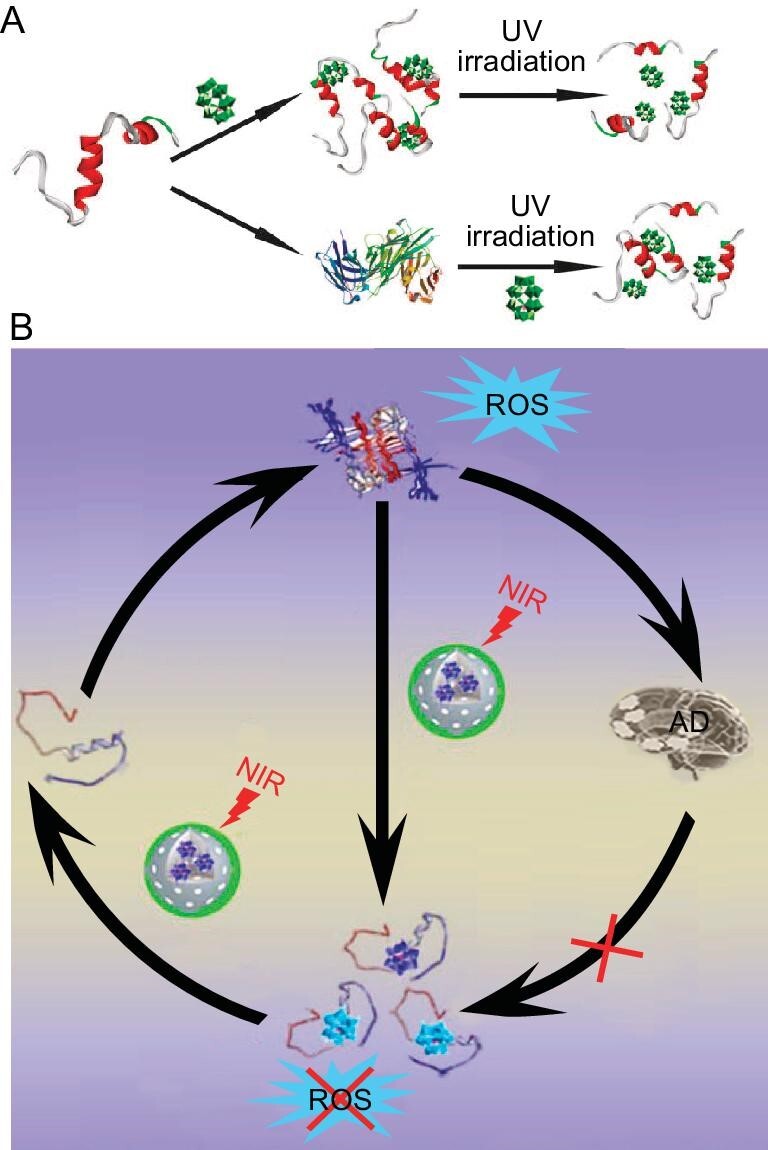
(A) Schematic illustration of POMs used to modulate Aβ aggregation upon UV irradiation. (B) Schematic drawing of redox-activated POMs for photothermal treatment of AD. Adapted with permission from ref. [71]; Royal Society of Chemistry; ref. [72], Wiley.

Because of its low invasiveness and high spatiotemporal controllability, phototherapy has been extensively explored for the treatment of various localized diseases involving pancreatic, prostate, and breast cancers [65,66]. However, phototherapy has been seldom considered for the application in neurodegenerative diseases [67,68]. POMs, which generally exhibit reversible multi-electron redox transitions, are very promising photocatalysts for diverse applications such as water splitting, pollutants degradation, and cancer therapy [34,69,70]. Inspired by the unique photocatalytic activities of POMs, Li *et al.* found that Wells-Dowson POMs K_8_[P_2_CoW_17_O_61_] displayed enhanced inhibition effects against Aβ self-assembly under UV irradiation (Fig. 9A), which was ascribed to the photodegradation of Aβ by the singlet oxygen (^1^O_2_) released from the photocatalyst K_8_[P_2_CoW_17_O_61_] [71]. The work presented here took a major step toward the development of AD phototherapy.

Ma *et al.* also prepared a series of redox-activated POMs with Keggin structure for photothermal treatment of AD (Fig. 9B) [72]. Reduced POMs (rPOMs) were nicely embedded in the mesoporous silica nanoparticles (MSNs). Then, the resulting rPOMs@MSNs were incorporated into thermo-responsive polymers for preventing the leakage of rPOMs. On one hand, under near-infrared (NIR) light irradiation, rPOMs could produce local hyperthermia to destroy mature Aβ fibrils. On the other hand, the external polymers were melted by the local heat responses, thereby releasing rPOMs. The released rPOMs not only hindered Aβ aggregation but also scavenged superfluous ROS. Intriguingly, among these used rPOMs, rPOM with Wells-Dawson structure showed a much better behaviour.

## POMS AS ARTIFICIAL ENZYMES/NANOZYMES FOR DEGRADING A**β**

**Figure 10.**
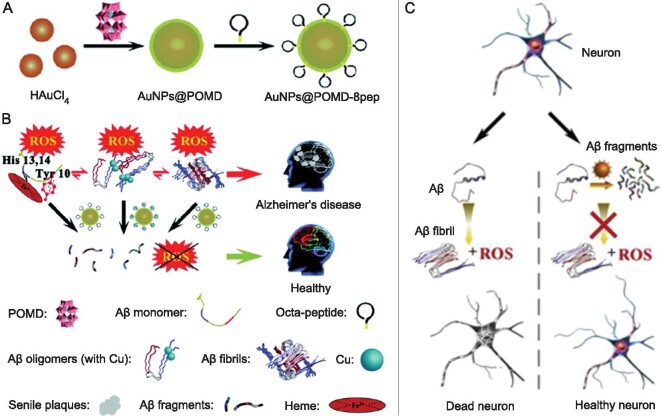
(A) Synthetic route of AuNPs@POMD-8pep. (B) AuNPs@POMD-8pep for the modulation of multiple facets of AD. (C) CeONP@POMs protected the neuron from cytotoxicity by synergy between Aβ degradation and ROS scavenging. Adapted with permission from ref. [79], Springer Nature Ltd; ref. [80], Elsevier.

A growing body of research reveals that the accumulation of brain amyloid plaques is not only correlated with Aβ generation, but also dependent on Aβ degradation. Proteolysis of Aβ, a very promising method to lower brain Aβ levels and to mitigate Aβ-mediated neurotoxicity, has gained much attention in recent years [73]. Currently, various natural enzymes, including insulin-degrading enzyme, endothelin converting enzyme, and neprilysin, have been developed for efficient Aβ hydrolysis [74,75]. However, the intrinsic limitations of natural enzymes, such as poor stability, high cost, and cumbersome purification, severely restrict their practical applications. In these contexts, Aβ-degrading artificial enzymes have emerged as a potential alternative to natural enzymes [76–78]. Recently, Gao *et al.* designed and synthesized a POMs-based artificial enzyme (denoted as AuNPs@POMD-8pep) for multi-faceted treatment of Aβ aggregates (Fig. 10A and B) [79]. The AuNPs@POM-8pep was composed of three ingredients: Wells-Dawson POM (POMD) that attacked the peptide bond, AuNPs that promoted electron transfer and increased the hydrolysis rate, and octa-peptide (N-Cys-His-Sar-His-Sar-His-Sar-His) (Fig. 10A). The resulting AuNPs@POMD-8pep possessed both high protease-like activity and superoxide dismutase (SOD)-like activity, which depleted Aβ aggregates and scavenged Aβ/Cu-induced ROS (Fig. 10B). Intriguingly, AuNPs@POMD-8pep could chelate Cu(II) ions and cut off Cu(II)-expedited Aβ aggregation. The AuNP@POMD-8pep was subsequently covalently functionalized with an Aβ-targeted peptide (CLPFFD), which could stem misdirected or undesirable proteolytic hydrolysis reactions. More importantly, the AuNPs@POMD-8pep efficiently crossed the BBB.

Guan *et al.* also constructed a series of Ceria/POMs nanohybrids (CeONP@POMs) with both robust protease-like activity for degrading Aβ peptides and high SOD-like activity for scavenging intracellular ROS (Fig. 10C) [80]. Among them, CeONP@POMD with Wells-Dawson type performed the highest hydrolysis activity toward Aβ. The CeONP@POMK with Keggin type showed moderate hydrolytic activity. The CeONP@POMA with Anderson type possessed the lowest activity. The CeONP@POMD not only facilitated PC12 cell proliferation, but also diminished BV2 microglial cell activation caused by Aβ. More intriguingly, CeONP@POM could cross the BBB and possess good biocompatibility.

## CHALLENGES AND OUTLOOK

POMs, a promising class of metallodrugs, are capable of effectively inhibiting Aβ aggregation. POMs are known to bind to Aβ mainly through size-dependent electrostatic interactions with positively charged His13-Lys16 (HHQK) regions of Aβ [44]. It is well established that the cationic cluster HHQK plays key roles in both Aβ oligomerization and fibril propagation. Thus, this binding could efficiently block Aβ conformational changes and shift the equilibrium away from fibrillization. There are numerous factors known to affect POM-Aβ interactions. Owing to the primarily electrostatic nature of the interactions, the POMs’ charge plays a pivotal role on its inhibition ability. Besides, the size of POMs also influences POM-Aβ interactions. For example, relatively large Wells-Dawson POMs exhibit a stronger inhibition effect than Keggin and Anderson types. Last but not least, the ability of POMs to gain access to the binding pocket in Aβ aggregates based on their shape can affect POM-Aβ interactions [52].

Although the inhibitory effect of POMs is quite promising, purely inorganic POMs exhibit limited Aβ selectivity. The functionalization of POMs offers a direct and highly controllable approach to modify both the structure and bioactivity of POMs. Enhanced POM-Aβ interaction can be achieved by (i) covalent/coordination modification with bioactive molecules and metal ions, or (ii) constructing POM-based nanocomposites. Collectively, the emergence of hybrid POMs offers a promising strategy for combining POMs with targeting moieties to achieve novel POM-based inhibitors with reduced toxicity and enhanced specificity.

POMs, featuring adjustable compositions and varied structures, have been used as electro/photocatalysts because of their unparalleled benefits, including quasi-semiconductor traits, exceptional redox and solution stability [30,34,69,70]. Moreover, POMs have gained considerable interest due to their attractiveness as building blocks for the construction of multi-functional nanocomposites [22,26]. In addition to inhibiting Aβ aggregation by direct binding to the HHKQ region, POM-based nanocomposites are known to act as photosensitizers for photo-induced inhibition of Aβ aggregation [71]. Moreover, POM-based nanocomposites with highly Lewis acidic metal centers exhibit excellent hydrolysis activity toward Aβ peptides [80]. To tackle poly-pathological features in AD, the rational design of multi-functional materials by integrating two or more therapeutic moieties into one nanocomposite has gained particular attention [4]. Taking advantage of the flexible structural modifiability of POM, designing multi-functional POM-based therapeutic agents is a promising strategy for AD therapy.

Although meticulously designed POM structures yield desired properties, the intricacy of POM systems presents considerable obstacles in comprehending POM-Aβ interactions under physiological conditions. This is because, in general, the POM-Aβ interactions involve multiple weak non-covalent interactions, including electrostatic interaction, hydrogen bond, and coordination interaction. Thus, POM-Aβ interactions are highly susceptible to environmental alterations, such as pH, temperature, and ionic strength [21,23,24,29–33,39,40,42]. In addition, the relatively low stability and suboptimal biocompatibility of POMs in physiological environments will hinder their application in AD therapy [38,81]. Moreover, the surface features of POMs limit their infiltration into cells, leading to potential cytotoxicity [40]. Hence, predicting and analyzing POM-protein interactions under physiological and pathological conditions, as well as exploring the mechanism of POM toxicity actions, are crucial challengs that need to be addressed, especially for the development of POM-based therapeutic agents.

Despite these extensive progresses, POMs as anti-amyloid agents are still in the infancy of development and there is a long way to go before clinical applications. Many issues still exist, such as insignificant BBB penetration, high cytotoxicity, and unclear biological and pharmacokinetic properties. Much more work is undoubtedly necessary to overcome those challenges and pave the way for POMs as the next generation of anti-AD metallodrugs under the interdisciplinary cooperation of researchers, such as structral biologists, medicinal chemists, biochemists, and neurologists. We are only at the dawn of POMs targeted toward the inhibition of Aβ aggregation, and expect to shed light on the developments of AD treatment in the near future.
